# Position statement on access to care in rare liver diseases: advancements of the European reference network (ERN) RARE-LIVER

**DOI:** 10.1186/s13023-019-1152-z

**Published:** 2019-07-08

**Authors:** Lucas H. P. Bernts, David E. J. Jones, Marleen M. Kaatee, Ansgar W. Lohse, Christoph Schramm, Ekkehard Sturm, Joost P. H. Drenth

**Affiliations:** 1 RARE-LIVER European Reference Network; Department of Gastroenterology and Hepatology, Radboud university medical centre, P.O. Box 9101, 6500 HB Nijmegen, the Netherlands; 2 RARE-LIVER European Reference Network, Institute of Cellular Medicine, Newcastle University, Newcastle upon Tyne, NE2 4HH UK; 3 PSC Patients Europe, P.O. Box 55, 2120 AB Bennebroek, Netherlands; 4 RARE-LIVER European Reference Network; Department of Medicine, University Medical Centre Hamburg-Eppendorf, Martinistraße 52, 20246 Hamburg, Germany; 5 RARE-LIVER European Reference Network; Department of Pediatric Gastroenterology and Hepatology, University Children’s Hospital, Calwerstraße, 72076 Tübingen, Germany

**Keywords:** European reference network, ERN, Rare liver disease, Autoimmune liver disease, Paediatric liver disease, Structural liver disease

## Abstract

The European Reference Network for rare liver diseases (ERN RARE-LIVER) is a Europe-wide network of paediatric and adult hepatologists from expert centres in close collaboration with patient advocates from the various disease-areas covered in our ERN. The ERN is focused on providing more equitable care across Europe and creates a network of both medical specialists and patient experts in rare liver disease. This position paper summarizes the achievements of the first year and plots the route for the near future for ERN RARE-LIVER, as discussed during a strategy meeting that took place 27 and 28 February 2018 in Nijmegen, the Netherlands. ERN RARE-LIVER has established itself as a group with experts, hospitals and patients. One of the tools to improve communication is the clinical patient management system (CPMS) that allows access to expert consultation by European physicians confronted with a patient with rare liver disease. ERN RARE-LIVER will function as the platform to improve healthcare by initiating registries, foster research efforts and coordinate development of clinical guidelines in Europe.

**Electronic supplementary material:**

The online version of this article (10.1186/s13023-019-1152-z) contains supplementary material, which is available to authorized users.

## Background

**Fig. 1:**
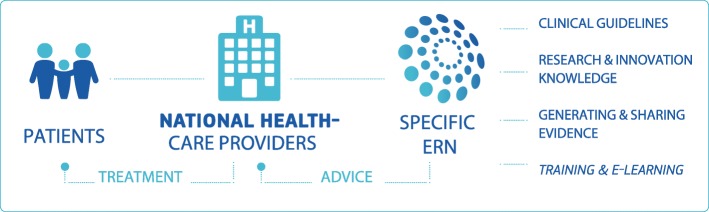
Goals of the European Reference Networks (ERNs). The Rare Disease Specific European Reference Networks (ERNs) are a project set up by the European Union to ensure more equitable care across Europe by creating networks for virtual expert consultation, knowledge generation and knowledge dissemination (Source: European Reference Network Brochure)

European Reference Networks (ERN) for rare diseases (Fig. 1) have been initiated by the European Commission as a means to achieve equitable care for rare diseases across Europe [1]. The ERN programme started in earnest in 2017 with the establishment of 24 ERNs within a European legal framework dedicated to rare or low prevalence complex diseases. This brought together more than 300 health care providers (hospitals) and 900 expert teams. The novelty of the programme offers a certain degree of freedom (and uncertainty) with respect to path and direction to take [2]. The vision of the establishment of ERNs was to realize more equitable access to the best healthcare across Europe and to drive improvement of standard of care and clinical knowledge of rare diseases in Europe. Working closely with the clinical centres, physicians and patients, the ERN must disseminate best clinical practice, use innovative IT solutions to enable clinicians to access expert knowledge across Europe, update clinical guidelines to enable standardisation of care and to provide patients with relevant high quality information.

### ERN RARE-LIVER

**Fig. 2:**
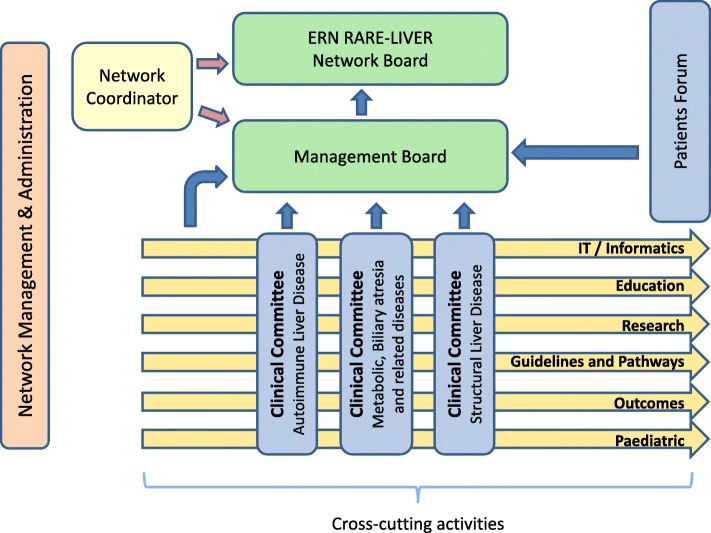
Governance structure. ERN RARE-LIVER comprises a network board and a management board directed by the network coordinator (currently Prof. Ansgar Lohse). The operational arm of the network is supported by three disease pillars. Pillar 1: Autoimmune liver disease. Pillar 2: Metabolic, Biliary atresia and related diseases and Pillar 3: Structural liver disease. Each pillar is directed by a clinical committee and is made up by experts from the health care professionals and patient advocates

The European Reference Network for rare liver diseases (ERN RARE-LIVER) has been established as a Europe-wide network of paediatric and adult hepatologists from expert centres with the aim to improve clinical management and research of rare liver disease in adults and children. The ERN currently covers paediatric and adult care for 12 different rare liver disorders, distributed over three pillars (Fig. 2). Pillar 1 (autoimmune liver disease) includes primary biliary cholangitis (PBC), autoimmune hepatitis (AIH), primary sclerosing cholangitis (PSC) and IgG4 disease. Pillar 2 (metabolic, biliary atresia and related diseases) includes alpha-1 anti-trypsin disease, Wilson’s disease, genetic cholestatic disease, biliary atresia and choledochal cysts. Pillar 3 (structural liver disease) includes polycystic liver disease (PLD) and other cystic liver disease, intrahepatic cholangiocarcinoma and vascular liver disease.

The ERN RARE-LIVER consists of 28 expert centres, and is gradually expanding (Additional file 1). Key to the success of ERN RARE-LIVER is close collaboration with patient organizations and groups, at the moment numbering 37.

There is an unmet need in the arena of rare liver disorders. Most rare liver disorders are infrequent and expertise is concentrated in few centres. Expert knowledge of rare liver diseases is scattered, concentrated with a few experts, takes many years to accumulate, and is bound to core facilities that allow knowledge building. Patients with rare diseases do not necessarily live close to an expert centre, certainly not when seen on a European scale. The vision of ERN RARE-LIVER is that patients with a rare liver disease deserve access to the best health care regardless of their geographical location. This vision centres on the realization that knowledge must travel and that the patient should be able to stay in his/her own healthcare environment. Locally applied expert knowledge not only directly benefits patients, but it also aids in dissemination of knowledge to local healthcare professionals and helps expert centres because they get access to patients and their disease behaviour which was impossible to get otherwise.

**Fig. 3:**
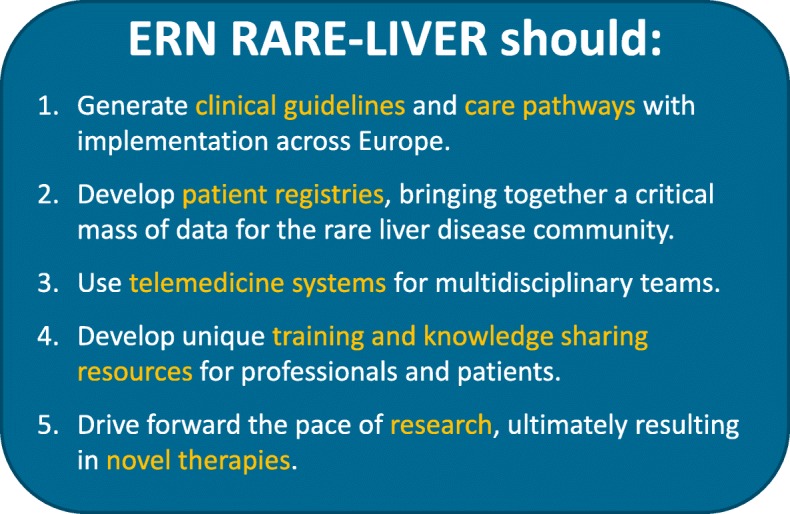
Goals of the ERN RARE-LIVER

An account of the foundation and the set goals of the ERN RARE-LIVER was published last year and the network broadly formulated five goals (Fig. 3) [3]. This position paper summarizes the achievements of the first year and plots the route for the near future for ERN RARE-LIVER, as discussed during a strategy meeting that took place 27 and 28 February 2018 in Nijmegen, the Netherlands. This was the 2nd meeting of the network after the inception meeting that took place on April 21, 2017 at the occasion of the European Association for the Study of the Liver (EASL) International Liver Congress in Amsterdam, the Netherlands.

### Achievements

The focus of Year 1 of the ERN RARE-LIVER was to get the organisational structures in place and secure funding for an elementary, yet efficient, framework. The map for the year ahead is to move from idea and governance structure to a lively and vibrant organisation that actually starts to deliver: making a difference for patients.

The network achieved to establish a governance framework and put a number of monitoring systems in place to gauge success such as a risk registry and a quality control database. A very important step entailed the survey of guidelines. ERN RARE-LIVER operates closely in cooperation with European Society for Paediatric Gastroenterology Hepatology and Nutrition (ESPHGAN) and EASL and both organisations are committed to assist physicians and other health care providers as well as patients and those interested in the clinical decision-making process by describing a range of generally accepted approaches for the diagnosis, treatment and prevention of complication of specific liver diseases. The data are critical to perform a gap analysis and identify disorders / clinical situations that are in need of a guideline.

## Plans & goals

The plans for the near future for the ERN RARE-LIVER fall in 3 categories.

### Things we have to do

As ERN we will have to report progress on the outcome parameters. To do so we will have to utilise the approved platforms such as the clinical patient management system (CPMS). In order to achieve a better reach in the European Union we must support new members.

### Things we need to do

The gap analysis is needed to identify clinical areas in need of guidelines or standards. This requires close collaboration with professional organisation such as EASL and ESPGHAN and other stakeholders [4]. We will need to participate in tele-boards to really act as a network of experts. A fine example comes from the Dutch Pancreatitis Study Group who has launched an online nationwide, multidisciplinary expert panel for clinicians treating patients with acute necrotizing pancreatitis. This panel helps in clinical decision making and has proved to be an accessible and valuable tool for treating clinicians [5].

### Things we want to do

ERN acts as a marketplace which we want to populate with clinicians interested in rare liver diseases. It offers us with an opportunity to meet and interchange with other professionals who have a shared interest in rare liver diseases. In order to improve clinical care we want to use the ERN as a network to facilitate, and direct clinical trials at a European scale. The patient advocates in our ERN are available to assist health care professionals where needed. Parallel to the activities of the health care professionals, they will focus on both training the patient advocacy community as well as incorporating the patient’s voice in the health care professionals’ trainings. Lastly, we are responsible for the clinicians of the future and we need to educate and train those clinicians who are able to provide excellent clinical care for the patients of tomorrow.

### Healthcare

The Clinical Patient Management System (CPMS) is essential for interaction between healthcare professionals and experts on clinical decision making, and was provided by the European Union to all ERNs. CPMS supports online multidisciplinary meetings (tele-boards) to discuss patients with diagnostic or therapeutic dilemmas in need for expert consultation. CPMS offers the opportunity to upload and share clinical data of patients and pictures such as histological slides or Magnetic Resonance Cholangio-Pancreaticography images. Importantly, this is fully in line with European data protection law. For example, a much debated issue during CPMS consultations revolves around AIH patients who have failed standard treatment and are in need of second or third line therapy [6, 7]. There is a wide variation in the management of difficult-to-treat AIH patients and although good quality evidence is lacking, there is considerable experience with different second and third line treatment at different ERN RARE-LIVER centres [8]. Tele-boards via CPMS and thereby expert consensus assist enormously in the clinical management of these patients [9].

In addition, CPMS is open to experts and advice-seeking health care providers outside ERN RARE-LIVER. It is possible to bring a difficult case to the expert panel via guest accounts to CPMS, even if the health care provider is not affiliated with a specialist centre of the ERN. For technical support, appointing to the experts in the field and to provide assistance wherever possible, a CPMS helpdesk has been installed by ERN RARE-LIVER at the University Medical Centre Hamburg-Eppendorf, Germany (CPMS.rareliver@uke.de). In addition, the EU (European Union) and the software provider of CPMS assure constant updates of CPMS, to simplify the access process, improve workflows and address needs of customization for the respective ERN.

### Transitional care

Transitioning adolescents and young adults with chronic rare liver diseases can be complex. [10] There is no uniform model for successful transition, but expert opinion suggests that a transition program enabling an increasing responsibility for health management is important. Expert recommendations support a transition process that starts in early adolescence, and provides continuous guidance and support. An excellent transition process allows for care opportunities with patients and caregivers, with the patient alone and between paediatric and adult professionals. We consider transition of care as a unique opportunity for ERN RARE-LIVER as the model that will be designed will benefit children with and without rare liver disorders but has the added benefit to bring paediatric and adult hepatologists closer together to improve this shortcoming that is also seen as problematic by patients and patient organizations. Specifically, it is our ambition to establish continuity-of-care pathways from paediatric to adult care in all ERN centres within the next two years. This should include the parents, who often feel lost when their child transitions to adult hepatology. Every child should be able to chart their individual transition to adult care.

### Outcome monitoring

Registries may be the answer to the lack of solid evidence in the field of rare liver disease [11]. By definition, a registry is an organized system that uses observational study methods to collect existing or uniform clinical data from individual patients. A registry offers a unique opportunity to conduct research on populations and conditions that are not generally studied in clinical trials, yet are important to clinical decision-makers. This is the reason why a prospective online registry has been developed by members of the ERN RARE-LIVER. We aim to include incident patients with a rare liver disease seen in one of the expert centres. In addition, we will include patients entered through CPMS who will be discussed during the tele-boards. Three benefits will emanate from this registry. In the first place, this registry will be used to assess quality aims that are set by each of the pillars. This will allow close monitoring of the quality aims and goals that have been set, by us, as ERN RARE-LIVER. Good examples for quality aims are: > 70% of patients achieve remission within 12 months of AIH onset, > 90% of primary PSC and PBC patients should have been queried about pruritus, and > 90% of PBC patients should receive the correct ursodeoxycholic acid dosage. For cystic liver diseases, we aim to include all new patients in the registry and enable integration with the previously established International PLD Registry [11]. From a research perspective, benefits of a prospective registry are that we might measure the natural clinical history as well as clinical effectiveness and provide follow up for delayed or long-term benefits or harm. Last but not least, the patient will benefit from the expertise and gains access to proper care. The ultimate goal is to improve quality of care for rare liver diseases. This is done by measuring what we do and what impact that has on disease outcomes.

**Fig. 4:**
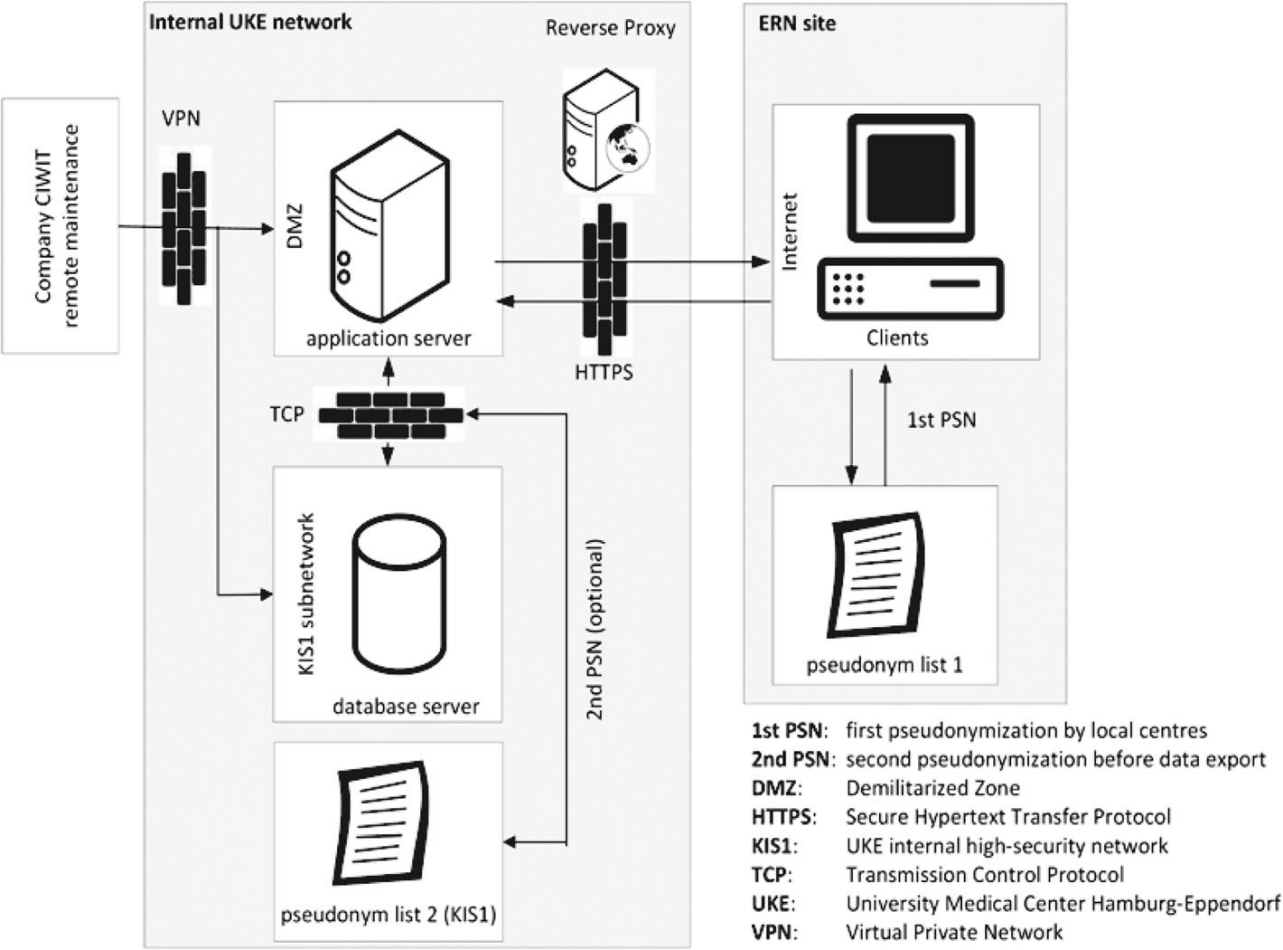
International registry data protection structure. We present the digital structure of the international registry, which will be used for outcome monitoring of quality aims and research questions. Each participating centre will retain ownership of the data. All data is uploaded pseudonymized. Privacy is secured according to German laws and guidelines, thus the system can be used Europe-wide. The versatile user-interface of the registry will run on Castor EDC (CIWIT B.V., Amsterdam, the Netherlands)

The EU General Data Protection Regulation (GDPR) is a new European law, effective 25 May 2018 that protects and empowers EU citizens data privacy and reshapes the way organizations across the region approach data privacy [12]. The conditions for consent have been strengthened, and consent forms must be clear and formulated in easily accessible terms and it must be as easy to withdraw consent as it is to give it. The prospective registry set up by the ERN, abides by the new privacy legislation and can be used in any EU member state (Fig. 4).

### Clinical care

As a consortium of the European experts on the subject of rare liver disease, the ERN sees it role fit to create position papers on subjects within rare liver disease where evidence is lacking, but advice for patients and clinicians is necessary. The gap analysis – in cooperation with patient organizations - will assist us in order to identify uncovered areas (which are likely to exist in rare liver disease). Subsequently we would target a number of areas where the development and implementation of standardised protocols and guidelines would most benefit patients with rare liver diseases. One example that is sought after by patients is a position paper on pregnancy in autoimmune liver disease.

The ERN will start to design a plan for guidelines and care pathways that can be made in association with EASL and ESPHGAN. This set of guidelines is key to the promise to offer patients the same chance for diagnosis and adequate treatment throughout Europe. In addition to developing guidelines, we are now looking at those already in existence; how we can endorse their content and how we can ensure that they reach the widest possible audience. We are designing a procedure that will help to endorse existing guidelines in collaboration with EASL and ESPGHAN in order to get the widest reach as possible. A key task of the ERN RARE-LIVER network will be the development of a care pathway to accompany these guidelines, which will outline the key steps in management and facilitate their delivery in practice across Europe [13].

We note that not all rare liver diseases are covered by our ERN. We are starting to map the route towards expansion of the number of diseases. This will be driven by the need of patients. For example, autosomal recessive polycystic kidney disease (ARPKD) is a disorder that presents in childhood with predominantly extra-hepatic features such as renal impairment and lung hypoplasia, only when children reach adulthood, liver related complications as a result of fibrosis formation start to ensue. Patient representatives have asked us to put this on our radar.

### Teaching and training

ERN RARE-LIVER will identify gaps within the current training of adult and paediatric hepatologists and aims to develop strategies to provide education, training and continuing professional development to address shortcomings. As such ERN RARE-LIVER strives as focal point for medical training and research, for both healthcare professionals and patient advocates. Few patients with a rare liver condition currently benefit from effective or curative therapies, and many do not have a definite diagnosis. Many have little easy-understandable knowledge of their disease in their native language and patient advocates can be helpful to fill that hole. Interweaving healthcare and innovative research helps to address this issue and is close to the heart of ERN. The combined expert centres have a large repository of teaching materials. The remit of ERN RARE-LIVER is to extend the reach of teaching documents. This is relevant for both patients and clinicians. The proposed ideas are: listing all the materials on the ERN RARE-LIVER website for easy access, creating e-learning modules, including content in curricula for fellows and exchange of knowledge between centres, for example with Marie Curie fellowships. In particular, ERN will also be producing patient summaries of guidelines, with the goal that patients can be offered a lay version, available in their native language. This will contribute to the goals set for the ERN. Finally, we plan to populate an ERN School for Rare Liver Diseases which helps to inform the wider scientific community about these diseases.

### Research

Research is close to the heart of the experts who populate ERN RARE-LIVER. Innovative research has the potential to change the lives of patients by providing them with new tools to improve diagnosis, care and even cure. Rare liver diseases are ill researched and most patient oriented studies are observational by nature. While this yields very relevant information about disease and/or disease behaviour it does not allow study of the effect of interventions. This requires well designed clinical trials. Biomedical industry has started to develop interest in rare liver disorders and the recent POISE trial examining the effect of obeticholic acid in PBC is a good example of how collaboration between academia and industry can pave the way towards innovative research [14]. ERN has a vested interest in supporting investigator initiated clinical trials. ERN RARE-LIVER provides researchers with the indispensable network and permits access to patients. We identified the need for a research strategy group or desk consisting of a representation of liver experts who can assist and direct us which research strategy to adopt (and support) and which applications we want to support.

### Expansion

ERN RARE-LIVER was initiated by a core group of experts with an interest in rare liver diseases. In order to get the best reach and traction in the European Union, expansion of the number of sites is mandatory. The way forward is to expand the ERN by including new expert centres, in particular from those coming from countries that have not been included before. There are different forms of membership within the ERN networks: full membership, and more importantly, affiliated or collaborative partnership. Centres that aspire to membership or partnership must bring a true added value to the ERN and must have documented expertise in at least one of the diseases that the ERN in question covers. Other criteria that have been set by ERN RARE-LIVER are: a minimum of 2 active CPMS users per centre, contribution of 5 CPMS cases per year and to enter at least 30 patients into the R-LIVER registry.

#### Full membership

Full Members have the full voting right for ERN internal decisions, they get access to funding or resources linked to ERN and they get access to a full user account for CPMS. Only centres from the EU can apply. There are two steps that have to be taken for full membership of an ERN; relevant hyperlinks to WebPages and contact information have been added to Additional file 2. First, support of an applying centre’s national health ministry is required. The “Board of Member States” (BoMS) is the oversight body for the EU and has representatives from all the health ministries for member states; the respective national board member should be the first contact (Additional file 2). The second step comprises the completion of an assessment process which includes a form, a self-assessment and supporting data and potentially a site audit. The final approval will take place by the BoMS. At present, the assessment process is still being finalized, however, the need for national approval will remain. So if you are considering full membership, seeking this approval is a concrete step you can take now.

#### Affiliated partnership

In order for ERNs to deliver genuine added value to all European Union Member States, legislation makes provision for ‘*Member States which do not have representation from a member within an approved ERN’* to participate through affiliated partnerships. Centres from a member state that does not currently have a full member and which are interested in Affiliated Partnership must be designated by their member state and need to contact the BoMS representative in the first instance. If the country has a full member then options are either Full Membership or Collaborative Partnership. Applications can be handed in now until the opening of the call for full members. Application forms are provided by the BoMS.

#### Collaborative partnership

The collaborative partnership is a looser form of membership which does not bring ERN Centre Status but allows members to develop links with some of the activities of the ERN. Centres from the EU, European Economic Area, and non-EU can apply. In addition, both centres from member states with or without a full member can apply as a collaborative partner of the ERN. This does not entail an EU process and is managed by the ERN RARE-LIVER itself. The ERN provides its own Collaborative Partnership form to centres interested in joining. Centres can apply at any time. Collaborative Partners have no voting right for ERN internal decisions, they do not get access to funding or resources linked to ERN and they need a guest user account for using CPMS.

## The role of patient organizations

As patient organizations are central to the concept of ERNs, they will shape ERN RARE-LIVER together with experts from the field as they are uniquely positioned to identify the unmet needs among patients. Each pillar has 2 patient advocates as member of the clinical committee in order to represent, inform and direct. Patient advocates come from 37 patient groups and our effort is key to bring patient organizations together, which enhances cross -fertilization. The patient organizations play an essential role in informing the public and most importantly patients in Europe. Indeed, clear communication is key to improving healthcare. Patient advocates receive training through ERN activities through workshops. Eurordis has also offered them a broad range of training, including webinars, meetings, capacity training, Summer school and Winter School. In effect, clinicians teach patients and vice versa. For example, patient group representatives can join courses to learn more about their disease and other rare liver diseases. We think that the role of the patients in setting an agenda for research, guidelines and information sources is central to the idea of ERN. One notable achievement is the patient involvement in the recent PBC clinical practice guideline of EASL where patients helped to identify important areas for improvement of liver care and shift focus to symptoms that are underappreciated, such as fatigue in PBC [15]. As patient organizations are also increasingly committed in research projects, they emphasized that researchers must pay serious attention to patient engagement and that there is ample room for improvement. Patients need mentoring and coaching and expert patients/carers need to be enabled. Patients are often not unwilling, but need more assistance in order to deliver.

## Conclusions

This meeting was the first in its kind and met with the expectations. ERN RARE-LIVER is established as a group and the investments put into collaboration starts to pay off. The year ahead will be the start of a true integral cooperation between many expert centres in Europe with a clear goal to improve healthcare, research and education related to rare liver diseases.

**Fig. 5:**
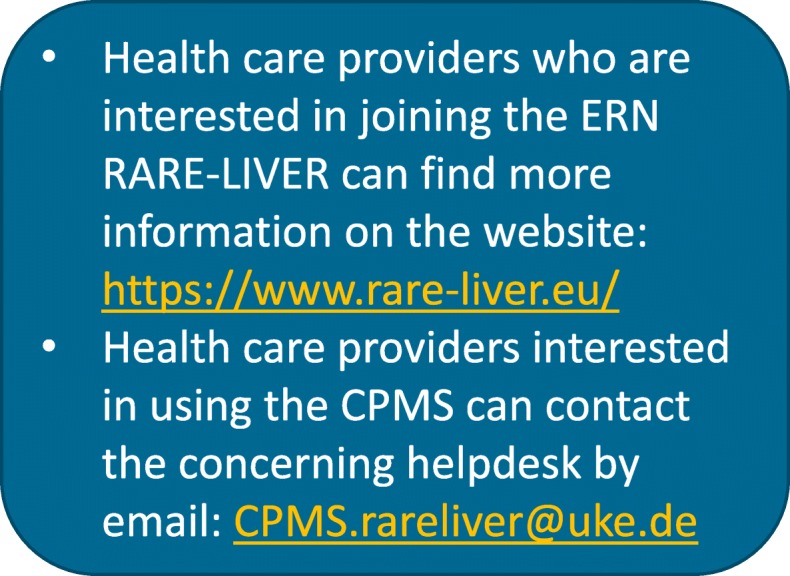
Seeking contact with ERN RARE-LIVER

Health care providers who are interested in joining ERN RARE-LIVER can find more information on the website: ‘https://www.rare-liver.eu/’, in Fig. 5, and Additional file 2.

## Additional files

Additional file 1: Collaborative Centres of the ERN RARE-LIVER (April 2019). (DOCX 46 kb)

Additional file 2: Important links (ERN RARE-LIVER). (DOCX 15 kb)

## Data Availability

Not applicable.

## Abbreviations

AIH: Autoimmune hepatitis
ARPKD: Autosomal recessive polycystic kidney disease
BoMS: Board of Member States
CPMS: Clinical patient management system
EASL: European Association for the Study of the Liver
EEA: European Economic Area
ERN: European Reference Network
ESPGHAN: European Society for Paediatric Gastroenterology Hepatology and Nutrition
EU: European Union
GDPR: General Data Protection Regulation
PBC: Primary Biliary Cholangitis
PLD: Polycystic liver disease
PSC: Primary Sclerosing Cholangitis
