# Supplementary material for: Position statement on access to care in rare liver diseases: advancements of the European reference network (ERN) RARE-LIVER
Source: Orphanet J Rare Dis. 2019 Jul 8;14:169. doi: 10.1186/s13023-019-1152-z (PMC6615270; doi:10.1186/s13023-019-1152-z)
Supplement: Supplementary file 1 — Collaborative Centres of the ERN RARE-LIVER (April 2019). (DOCX 46 kb) [file 13023_2019_1152_MOESM1_ESM.docx]

**Collaborative Centres of the ERN RARE-LIVER (April 2019)**

| 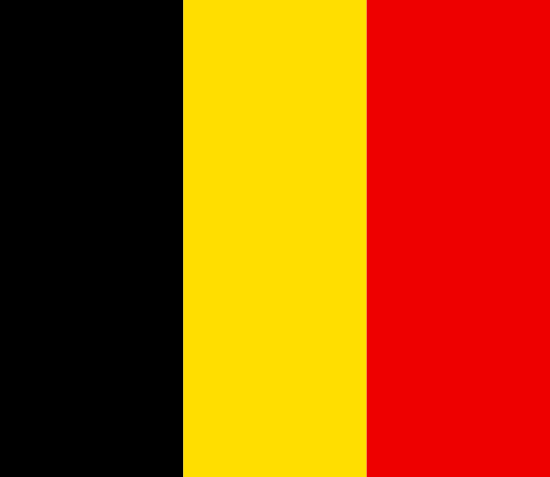  Belgium | 1. Université Catholique de Louvain (St Luc) 2. University Hospitals KU Leuven 3. University Hospital Ghent |
| --- | --- |
| 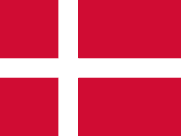  Denmark | 1. Copenhagen University Hospital Rigshospitalet |
| 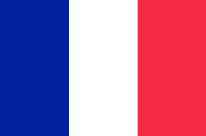  France | 1. Assistance Publique-Hôpitaux de Paris, Hôpital Bicêtre 2. Assistance Publique-Hôpitaux de Paris, Hôpital Saint-Antoine 3. Assistance Publique-Hôpitaux de Paris, Hôpital Beaujon |
| 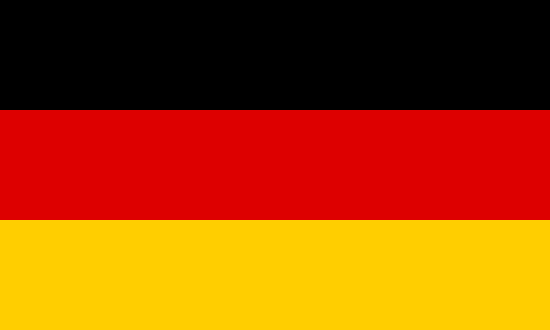  Germany | 1. Medizinische Hochschule Hannover 2. Universitätsklinikum des Saarlandes, Homburg 3. Universitätsklinikum Tübingen 4. Uniklinik RWTH Aachen 5. Universitätsklinikum Hamburg-Eppendorf |
| 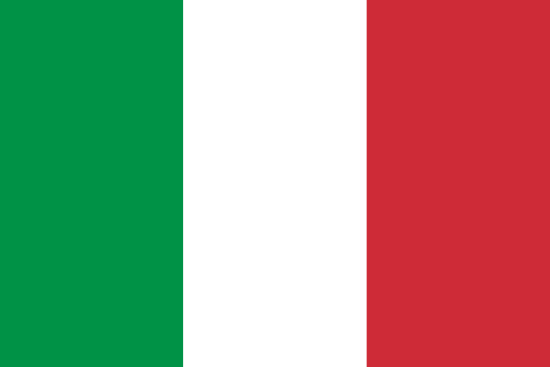  Italy | 1. ASST Monza, Ospedale San Gerardo 2. ASST Milan, Ospedale Santi Paolo e Carlo 3. Azienda Ospedaliera - Universitaria di Padova |
| 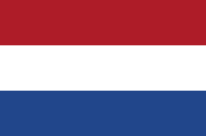  Netherlands | 1. Academic Medical Center Amsterdam 2. University Medical Center Groningen 3. Radboud University Medical Center Nijmegen |
| 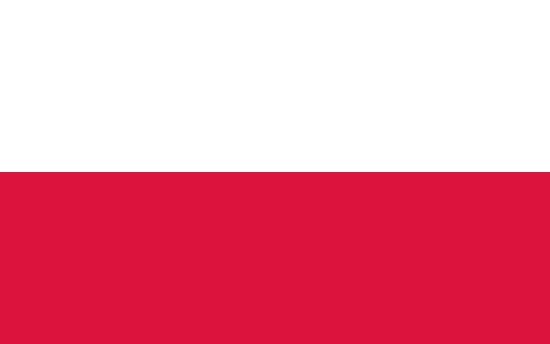  Poland | 1. Medical University of Warsaw |
| 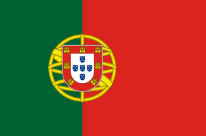  Portugal | 1. Centro Hospitalar e Universitário de Coimbra, EPE |
| 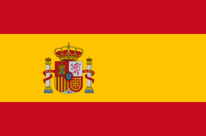  Spain | 1. Hospital Clínic i Provincial de Barcelona 2. Hospital Universitario "La Paz", Madrid |
| 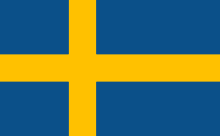  Sweden | 1. Karolinska University Hospital, Stockholm 2. Sahlgrenska University Hospital, Gothenburg |
| 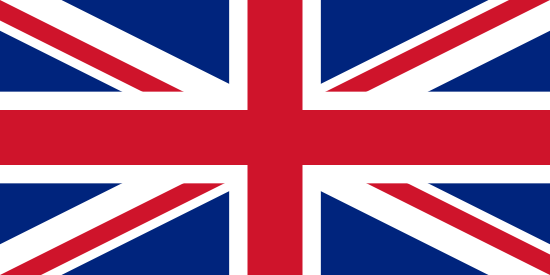  United Kingdom | 1. The Newcastle upon Tyne Hospitals NHS Foundation Trust 2. Royal Free London NHS Foundation Trust 3. University Hospitals Birmingham NHS Foundation Trust 4. Birmingham Children's Hospital NHS Foundation Trust |

**Planned affiliated partners of the ERN RARE-LIVER (April 2019):**

| 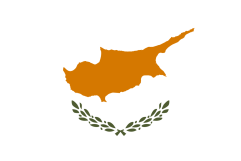 Cyprus | 1. Archbishop Makarios III Hospital and King's 2. College Hospital, Nicosia |
| --- | --- |
| 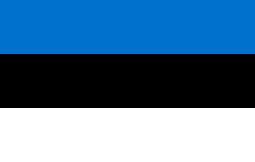 Estonia | 1. Tartu University Hospital |
| 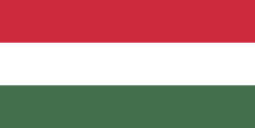 Hungary | 1. Clinical Centre of University of Debrecen |
| 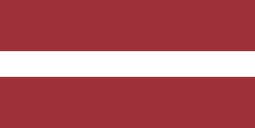 Latvia | 1. Clinical Children`s University Hospital Riga 2. Riga East University Hospital |
| 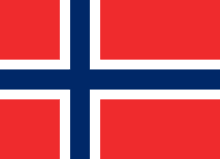 Norway | 1. Oslo University Hospital |
