# Supplementary material for: Position statement on access to care in rare liver diseases: advancements of the European reference network (ERN) RARE-LIVER
Source: Orphanet J Rare Dis. 2019 Jul 8;14:169. doi: 10.1186/s13023-019-1152-z (PMC6615270; doi:10.1186/s13023-019-1152-z)
Supplement: Supplementary file 2 — Important links (ERN RARE-LIVER). (DOCX 15 kb) [file 13023_2019_1152_MOESM2_ESM.docx]

**Important links:**

**European Reference Networks - Overview:**<https://ec.europa.eu/health/ern_en>

**Frequently asked questions:**
<https://ec.europa.eu/health/ern/implementation/faq_en>

**Members of the ERN Board of Member States (BoMS):**

<https://ec.europa.eu/health/sites/health/files/ern/docs/ern_board_members_en.pdf>

**ERN RARE-LIVER:**https://www.rare-liver.eu/

**Clinical Patient Management System (CPMS):**<https://ern-euro-nmd.eu/clinical-patient-management-system/>

**CPMS for ERN RARE-LIVER:**
Email address: CPMS.rareliver@uke.de
